# A comprehensive framework for considering additional unintended consequences in economic evaluation

**DOI:** 10.1186/s12962-020-00218-8

**Published:** 2020-08-04

**Authors:** Liv Nymark, Anna Vassall

**Affiliations:** 1 Department of Global Health, The University of Amsterdam and the Academic Medical Center (AMC), Meibergdreef 9, 1105 AZ Amsterdam, The Netherlands; 2 Department of Global Health and Development, London School of Hygiene and Tropical Medicine, London, WC1E 7HT UK

**Keywords:** Cost-effectiveness analysis, Indirect effects, Consequences, Externality, Internality, Guidelines

## Abstract

**Background:**

In recent years there has been a growth in economic evaluations that consider indirect health benefits to populations due to advances in mathematical modeling. In addition, economic evaluations guidelines have suggested the inclusion of impact inventories to include non-health direct and indirect consequences. We aim to bring together this literature, together with the broader literature on internalities and externalities to propose a comprehensive approach for analysts to identify and characterize all unintended consequences in economic evaluations.

**Methods:**

We present a framework to assist analysts identify and characterize additional costs and effects beyond that of direct health impact primarily intended to be influenced by the intervention/technology. We build on previous checklists to provide analysts with a comprehensive framework to justify the inclusion or exclusion of effects, supporting the use of current guidelines, to ensure any unintended effects are considered. We illustrate this framework with examples from immunization. These were identified from a previous systematic review, PhD thesis work, and general search scoping in PubMed databases.

**Results:**

We present a comprehensive framework to consider additional consequences, exemplified by types and categories. We bring this and other guidance together to assist analysts identify possible unintended consequences whether taking a provider or societal perspective.

**Conclusions:**

Although there are many challenges ahead to standardize the inclusion of additional consequences in economic evaluation, we hope by moving beyond generic statements to reporting against a comprehensive framework of additional effects we can support further consistency in this aspect of cost-effectiveness analysis going forward.

## Background

Guidelines for performing economic evaluations of healthcare interventions have emerged as a tool to support the quality and standardization of cost-effectiveness analysis [1–3]. Generally, these guidelines state that analysts should consider all relevant direct and indirect effects [4, 5], although these may be limited by the payer perspective, and often are contained to health effects. Some guidelines provide additional guidance on the inclusion of non-health effects [6, 7]. For example, the Second Panel on Cost-Effectiveness in Health and Medicine defines societal benefits as non-health effects and costs which are not accrued to health budgets, and provides a reporting checklist, known as an ‘impact inventory’ to report these. The checklist includes impacts on areas such as educational outcomes. The International Society for Pharmacoeconomics and Outcomes Research (ISPOR) has also highlighted the importance of mapping indirect health and non-health effects into economics frameworks for value assessment. The ISPOR Special Task Force report states that while indirect effects such as labor productivity and adherence improving factors are covered to some extent in current cost-effectiveness analyses, other categories such as scientific spillover effects are virtually absent [8]. Finally, various infectious disease-specific guidelines address the inclusion of non-direct effects, and some (for example, immunization) have made also attempts at scoping possible types of impact [9–11].

Despite this guidance, it can be challenging for analysts to comprehensively map out which non-direct health and non-health effects to include in analyses, in addition to the primary intended effect [12–14]. As many as two-thirds of cost-effectiveness studies do not consider how to include indirect health effects even when they may be substantial [15, 16]. We present here a framework to assist analysts identify and characterize additional costs and effects beyond that of direct health impact primarily intended to be influenced by the intervention/technology. Our aim is not to argue that all these effects should be included in every economic evaluation; this should depend on the perspective of the evaluation and strength of evidence supporting importance of the effect. Instead we aim to build on previous checklists to provide analysts with a comprehensive framework to justify the inclusion or exclusion of effects, supporting the use of current guidelines, to ensure any unintended effects are considered. We illustrate this framework with examples from immunization (see Table 1). These were identified from a previous systematic review [15], PhD thesis work [42], and general search scoping in PubMed databases.

**Table 1:** Examples of ‘internal’ and ‘external’ person consequences using immunization(s)

Perspective	Type	‘Internal’	‘External’	Example
Health effects	Biology	*Non*-*specific effects*		A marked reduced risk of dying from sepsis and pneumonia was observed in low-birthweight neonates who received BCG immunization at birth in an RCT in Guinea-Bissau [22]
		*Non*-*specific effects*		In a Dutch randomized placebo-controlled human challenge study BCG vaccination was found to induce genome-wide epigenetic reprograming of monocytes and protected against experimental infection with an attenuated yellow fever virus vaccine strain [23]
			***Transmission***	Marked declines in the diagnoses of genital warts in young Australian Aboriginal and Torres Strait Islander (Indigenous) men in Australia after the introduction of the HPV bivalent vaccination programme for females suggests that female vaccination offered indirect herd protection for men [25]
		*Pathogen response*		The change in patterns of serotype replacement or shifting causing invasive pneumococcal disease (IPD) produced marked declines in the incidence of IPD in children and moderate declines in adults following the replacement of the PCV7 vaccine by the PCV13 vaccine in Spain [27]
		*Pathogen response*		A comparison of the impact against IPD in PCV10 and PCV13 vaccinated counties in Sweden observed an increase in serotype 6C in PCV10 counties but not in PCV13 counties. This was assumed to indicate that serotype 6A, which is included in PCV13 but not in PCV10, offers carry-over protective effects against serotype 6C [28]
	Demand-side	*Change in health behaviour*		A universal immunization programme in Nepal (which aside from providing childhood vaccinations) also educates mothers on vaccination has been found to result in positive changes in other individual health behaviours, such as hygiene and sanitation practices and behaviours [34]
		*Change in health services consumption*		Following maternal tetanus vaccination in Bangladesh the consumption of health care services was markedly reduced indicating a positive change in the need to seek health care services [35]
	Supply-side		***Health Systems***	Hib vaccination prevents disease and thus reduces the need for antibiotic use for treatment which in turn reduces the development of antimicrobial resistance (secondary effects) [30]
Non-health effects	Demand-side	*Education*		PCV vaccination prevents pneumococcal pneumonia in children in South Africa. This has been associated with children improving their educational attainment [9]
		*Productivity*		Hib vaccination reduces child mortality. This allows mothers of vaccinated children achieve their target family size through fewer births. This has been shown to result in increased adult labour productivity for women [10]
			***Intra household***	A study from Belgium suggests that the caregiver burden within households in which children were not vaccinated against rotavirus was markedly severe; especially if no medical care was sought [11]
		*Intra household*		In Argentina, households have reported negative changes in behaviours when a member in the household has cervical cancer. This resulted in negative impact on educational attainment which could have been prevented with the HPV vaccination [12]
	Supply-side		***Outside health systems***	Yearly influenza immunization reduces infection and related complications in the elderly and therefore result in reduced use of social support services outside the health care sector, such as social care services [13]
		*Provider*		Previously influenza vaccinated healthcare providers demonstrated positive behavioural changes in their willingness to be vaccinated in forthcoming seasons [14]

Italic = internal person (host); bolditalic = external person (host)

*PCV* Pneumococcal conjugate vaccine, *BCG* Bacillus Calmette–Guérin, *Hib* Haemophilus influenzae type b, *HPV* Human papillomavirus vaccine, *RCT* Randomized Control Trial

## Methods

### Search strategy used to illustrate the framework

The immunization examples used to illustrate the proposed framework were identified from a previous review on cost-effectiveness analyses of human vaccines. Nymark et al. [15] utilized the following search strategy which they limited to English language with free text and MeSH terms; vaccin∗, economic evaluat∗, humans.

Other examples of unintended consequences of immunization were identified from a PhD thesis cost-effectiveness analysis of Bacillus Calmette–Guérin (BCG) versus no BCG vaccination for prevention of atopic dermatitis in neonates with predisposition to atopic disease [42].

Finally, a search scoping strategy which consisted of search terms ‘cost–benefit analysis’ or ‘cost–benefit’ and ‘externality’ was utilized to identify any additional examples of unintended consequences of immunization to illustrate the framework.

### Development and validation of the framework

The framework was developed following a review of current economic evaluations guidelines [1–11] and reviews of the economic evaluation for TB and immunization—in which relevant unintended consequences were identified and classified into different types; biology, demand and supply. Unintended consequences were structured based on existing frameworks such as the Second Panel on Cost-Effectiveness in Health and Medicine and ISPOR guidelines and then generalize commonalities among secondary effects from these.

Additional unintended consequences were also identified previous PhD thesis work on immunization [42].

### Types and categories of ‘internal’ and ‘external’ person consequences

Interventions can have impact beyond the intended direct health consequences; such as ‘internal’ consequences (occurring ‘within’ in the individual) and ‘external’ consequences (when the reaction occurs ‘outside’ the individual). We refer to the former as an ‘internality’ and the latter is commonly referred to in economics as an externalit*y*; defined as a cost or benefit which is caused by one person’s action while impacting others who are not part of the action [17]. In non-economic literature the term externalities is often used interchangeably in reference to indirect effects, unintended consequences, spillover effects and non-direct effects.

**Fig. 1:**
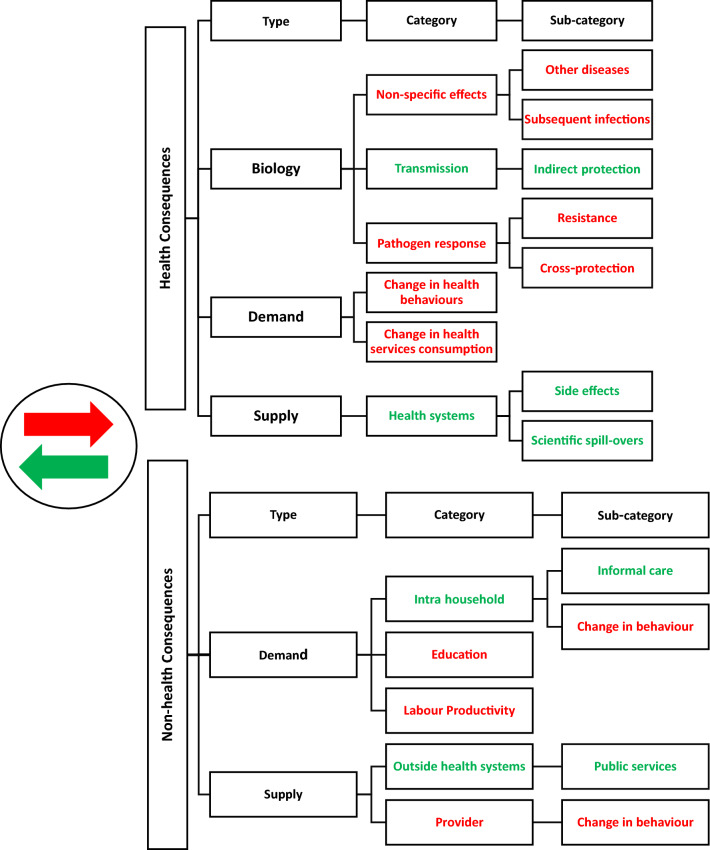
Conceptual framework ‘internal’ and’external’ consequences

Depending on perspective, analysts may wish to consider both additional health and non-health consequences. Within health impact, there are several types of ‘internalities’ and ‘externalities’ (see Fig. 1). These can be divided into biological effects and demand-side and supply-side behavioral consequences. The first type ‘biological’ includes the impact of the intervention on other diseases, infection and pathogens. Interventions can also impact individual, household and population health related consumption; ‘demand-side’ impact. Finally, interventions can also impact the supply side, by changing the behavior of health providers and impacting other health services or the provision of non-health services. Likewise, there may be internal and external non-health consequences demand and supply side impact. Figure 1 illustrates our framework and we provide further explanation and an example below.

### Health sector perspective

#### Additional biological effects

Biology refers to any chemical, molecular, cellular, immune or physiologic process or mechanism which is activated inside the human body [18]. Central to the description of the type biology are the functions and mechanisms which work within the human body and relate to how organisms, organ systems, cells and molecules carry out their chemical and physical functions and how these interactions are regulated, including control mechanisms and communication between cells [19]. For the type ‘biology’ there are three categories of potential additional ‘internal’ and ‘external’ person (host) effects. These are: (1) Non-specific effects (NSE) (impacts on other diseases); (2) transmission effects (infection to others) and (3) pathogen response (impact on the pathogen, such as creating new resistant disease).

An example of the first category (NSE) is the case of the BCG vaccine is indicated for the prevention of tuberculosis (TB) [20]. NSE of immunization are beneficial effects which offer protection beyond specific pathogens [21]. A study of BCG immunization at birth in low-birth weight neonates (< 2500 g) in Guinea-Bissau found a reduced risk of sepsis and pneumonia (non-specific diseases which are unrelated to the direct proportion which BCG offers against tuberculosis) (see Table 1) [22]. Likewise, BCG vaccine has been observed to train the adaptive immune response to become more vigilant to subsequent vaccinations [23]. Other examples of internal additional health effects include the impact of anti-retrovirals for HIV on the development of active TB from infection. NSE can be divided into two sub-categories, those that have consequence for other diseases and those that impact the likelihood of subsequent infections.

The second category, transmission is defined as the passing of an infectious agent (pathogen) from an infected host individual to another individual. The externality of transmission is increasing being included in economic evaluation of infectious diseases. In addition, the transmission of disease from human to human is reduced by immunization. Herd immunity is an indirect or ‘external’ person effect associated with immunization which occurs when unvaccinated (susceptible) individuals avoid infectious diseases because a large enough percentage of the population who surround them are immunized [24]. Thereby providing a measure of indirect protection for individuals who are not immunized. An example of herd immunity is the HPV vaccination programme for females in Australia which is associated with declines in the diagnoses of genital warts in young indigenous men in Australia [25]. Hence, vaccinated females not only reduced their own direct susceptibility but also reduced the risk for unvaccinated males who benefited from a measure of indirect herd protection even though they were not the direct recipients of the HPV vaccine.

The third category of biology is ‘pathogen response’ which refers to the human body’s reaction when an infectious agent (pathogen) causes disease or illness to its host. The category is classified as an ‘internal’ additional effect. In the case of immunization, vaccination can result in changes in the underlying serotype of the pathogen (in the case of other diseases, incomplete intervention can also result in resistance). Serotype is the term used to refer to a group of organisms within a species that have the same type and number of surface antigens. Antigens are molecules that are capable of stimulating an immune response specifically activating the body’s infection-fighting white blood cells. Serotype replacement is a phenomenon which induces resistance to sub-types of serotypes if the frequency of a sub-type of serotype declines due to high levels of immunity, allowing other serotypes to replace it [26]. Widespread use of pneumococcal conjugate vaccines has been associated with serotype replacement or shifting. As demonstrated in Navarra, Spain, the incidence of invasive pneumococcal disease (IPD) from serotypes not included in the PCV7 vaccine increased due to resistance following the introduction of this vaccine, which was attributed to serotype replacement. However, the incidence of IPD declined, and the change in patterns of serotypes causing IPD produced marked declines in children and moderate declines in adults following the replacement of the PCV7 vaccine by the PCV13 vaccine [27].

Another potential additional pathogen response is cross-protection. Cross-protection is the protection conferred on a host (‘internal’) by infection with one strain of a virus that prevents infection by a closely related sub-strain of that virus. Serotype cross-over effects are an example of this. In Sweden, a direct comparison of the impact against IPD was performed in 21 counties who used either PCV10 or PCV13 vaccination. An increase in serotype 6C in the PCV10 counties was observed but not in the PCV13 counties. This was assumed to suggest that serotype 6A, which is included in PCV13 but not in PCV10, offers cross-protection against serotype 6C [28].

Finally, pathogens can induce side effects. A side effect refers to an effect, whether therapeutic or adverse, that is secondary to the one intended [29]. Although the term is predominantly employed to describe adverse effects, it can also apply to unintended consequences of the use of a drug. An example of this is the Hemophilus influenzae type B (Hib) vaccination that by preventing bacterial infections from occurring or spreading prevents infections and thus reduces the use of antibiotic treatment that would have been used to treat infections. Subsequently this reduces the development of secondary effects such as antimicrobial resistance [30].

#### Additional demand-side consequences

The law of supply and demand is a theory that explains how the market allocates resources based on the interaction between the sellers of a resource and the buyers of that resource [31]. The demand-side refers to the demand of goods and services which individuals want to buy or consume [32]. Previous frameworks have outlined the type of demand and supply constraints that may be considered in economic evaluations in health sector [33]. In terms of additional consequences, the type demand-side, there are two categories related to health: Changes in health influencing behavior and changes in health services consumption (both ‘internalities’).

##### Changes in health behavior and health services consumption

Health behaviour refers to a person’s beliefs and actions regarding their health and well-being. An example of a positive additional behaviour consequences was observed in Nepal, where changes in mothers’ behaviours and knowledge regarding hygiene and sanitation practices have been observed as a consequence of a universal immunisation programme which educates mothers about vaccination [34]. Health care consumption refers to the utilization of additional (or reduction in utilization of) services by persons (‘internal’ person) for the purpose of preventing and curing health problems, promoting maintenance of health and well-being, or obtaining information about one’s health status and prognosis. For example, following maternal tetanus vaccination in Bangladesh the consumption of health care services (‘internal’ person) was markedly reduced indicating a positive change in the need to seek health care services [35].

#### Additional supply-side consequences

The supply-side refers to the part of the economy which involves the production of goods or supplying of services which are available to consumers or individuals [32]. For the type supply-side, we identify one category that fall under the health sector perspective: health systems (‘external’).

##### Health systems

Providing an intervention can have many positive and negative spillovers in the health system. In addition, the direct opportunity cost of spending on interventions, the way they are delivered may influence the quality and delivery of other health services. For example, training provided as part of the scale-up of immunization programmes, may strengthen general clinical and non-clinical skills, that may have positive benefits for the delivery of other services. A further example the benefits of influenza vaccination of healthcare workers is important for protecting staff and patients, for example previously influenza vaccinated healthcare providers demonstrated positive behavioral changes in their willingness to be vaccinated in forthcoming seasons thus generating additional benefit to the initial vaccination [36].

Finally, in recent years there has been some attention to the potential, particularly of new interventions, to have spillover scientific benefits for the development of other health related technologies. For example, the knowledge of the mechanism of action associated with a new a drug or vaccine might offer value beyond itself and lead to later innovations in drug development or the treatment of very different diseases. The BCG vaccine exemplifies this by subsequent discovery resulting in treatment possibilities aside from those originally indicated (see Table 1).

### Societal perspective

There is a wide literature of the possible non-health impacts of health interventions. These impacts can occur at the individual, household, and have follow-on consequences for the sectoral and macro-level.

#### Demand-side consequences

For the type demand-side, we highlight two broad categories: behavior/education/knowledge (‘internal’ person), and consumption of non-health goods.

##### Non-health changes in behavior and consumption

Health intervention can impact non-health related behaviors in many ways. One example is the impact on health on education. Education is defined as the process of learning and acquisition of knowledge, skills, values, beliefs, and habits. An illustration of how immunization enhances learning and the acquisition of knowledge (education) ‘external’ to the individual originates from South Africa. The pneumococcal conjugate vaccines have been associated with children improving their educational attainment, due largely to the prevention of pneumococcal pneumonia [14]. Changes in educational attainment may have follow-on consequences for a wide range of behaviors that impact social welfare.

Health intervention can also result in changes of consumption of non-health goods Households have reported negative changes in behaviours such as food consumption and school attendance in children when a parent in the household has cervical cancer. This resulted in negative impact on educational attainment for children in the household which could have been prevented with the HPV vaccination [37].

#### Supply-side consequences

For the type supply-side, we identify two categories that fall under the health consequences perspective: labor productivity (‘internal’ person) and provision of non-health services.

##### Productivity

Productivity impact is commonly captured in a societal perspective (although typically considered as a cost, rather than on the impact side of the incremental cost-effectiveness ration). In respect of immunization, the Hib vaccination has been demonstrated to increase mothers’ productivity in the labor market [38]. Hib vaccination can reduce child mortality resulting in mothers of vaccinated children can achieve their target family size through fewer births. Having fewer children, may enable mothers to invest more resources in their own development which results in increased individual productivity (‘internal’ person).

There may also changes in productivity in respect of informal labour. Informal care is generally defined as the unpaid care provided to older and dependent persons by a person with whom they have a social relationship, such as a spouse, parent, child, other relative, neighbour, friend or other non-kin [39]. An illustration of this comes from a Belgium study in which the caregiver burden within households in which children were not vaccinated against rotavirus was markedly severe; especially if no medical care was sought [40].

##### Non-health sector service provision

Non-health sector public service provision (‘external’) include areas like (e.g. public safety, infrastructure and social support services). An example of how immunization impacts services external to the healthcare system is yearly influenza immunization among the elderly. This reduces infection and related complications in the elderly and therefore results in reduced use of long-term social support systems outside the health care sector, such as institutional care in residential care home [41]. A simple checklist addresses (Table 2) how these categories of the effects of health technologies can be measured (tangible) or not (intangible) and what may condition their classification and inclusion into the economic evaluations.

**Table 2:** Checklist ‘internal’ and ‘external’ person consequences

	Type	Category	Yes	No	Sub-category	Yes	No	Condition	Yes	No
Health consequences	Biology	*Non*-*specific effects*			*Other diseases*					
					*Subsequent infections*					
		*Transmission*			*Indirect protection*					
		*Pathogen response*			*Resistance*					
					*Cross*-*protection*					
	Demand	***Change in health behaviours***								
		***Change in health consumption***								
	Supply	*Health systems*			*Side effects*					
					***Scientific spill*-*overs***					
Non-health consequences	Demand	*Intra household*			*Informal care*					
					***Change in behaviour***					
		***Education***								
		*Labour Productivity*								
	Supply	***Outside health systems***			***Public services***					
		***Provider***			***Change in behaviour***					

Italic = Tangible unintended consequences

Bolditalic = Intangible unintended consequences

## Discussion and conclusion

We present a comprehensive framework to consider additional consequences. While there is other guidance, we bring this guidance together to assist analysts identify possible unintended consequences whether taking a provider or societal perspective. There is currently a rigorous debate around capturing the full value of health interventions both within and beyond the health sector. The intention of this paper is not to support the inclusion of all additional consequences in every economic evaluation, but instead provide a framework to identify those that may be important. Moreover, any effects identified may not be additive in terms of overall value. Many of the effects listed interact (also with intended direct health effect) and care should be taken to also identify any interactions, at the individual, household, sectoral or macro-level.

The consideration of additional effects can influence the analytic choice of health outcome measures used, and non-health outcome measures included. For example, vaccine-induced improvements to immunity against non-targeted antigens (i.e. ‘internal’ person effects) manifest in non-specific epidemiological health outcomes, such as for example all-cause acute gastroenteritis related hospitalizations that may not typically measure in vaccine trials.

In addition, it may impact other methodological choices. For example, if the framework identifies a potentially major non-specific effect, it may be important to identify a comparator that also addresses that effect in the economic evaluation. For example, if the effect of the BCG vaccine extends beyond the protection against TB, and has substantial effects on auto-immune conditions (e.g. atopic dermatitis in high risk infants) [42], an economic evaluation could consider comparing interventions or strategies beyond TB. Furthermore, the analytical choice of target population and subgroups for the economic evaluation may also change when considering non-direct effects. The additional effects of the BCG vaccine, as described above, may result in the comparison of the existing strategy of BCG vaccination to a strategy in which subgroups or risk groups who are not recommended to receive the vaccine on grounds of risk of TB are included.

Applying this framework will not be without empirical challenges. Firstly, once potential additional consequences are identified, their importance will need to be assessed. Determining when a consequence is sufficiently important is often challenging a priori and will be based on a mixture of prior evidence of plausible effect. Moreover, even where there is evidence from other populations, the data to parameterize additional consequences may not be available for the population being evaluated. Further guidance is therefore required as to how to make the decision to include an additional consequence identified or when to simply report it qualitatively (as in the Second Panel impact inventory). While guidelines are clear that analyst should include all costs and effects relevant to the study perspective, practice on which are included varies substantially, which may lead to inconsistent decisions. Although there are many challenges ahead to standardize the inclusion of additional consequences in economic evaluation, we hope by moving beyond generic statements to reporting against a comprehensive framework of types and categories of additional effects we can support further consistency in this aspect cost-effectiveness analysis going forward.

## Data Availability

Secondary data and material only
